# Gastric metastases of oral carcinoma resulting from percutaneous endoscopic gastrostomy placement via the introducer technique

**DOI:** 10.1093/gastro/got027

**Published:** 2013-10-15

**Authors:** Jun Liang Teh, Reuben K. Wong, Michelle Gowans, Asim Shabbir, Bhavesh Doshi, David E. Ong, Victor T. Fan

**Affiliations:** ^1^Division of General Surgery, National University Hospital, Singapore, ^2^Division of Gastroenterology & Hepatology, National University Hospital, Singapore and ^3^Department of Oral and Maxillofacial Surgery, National University Hospital, Singapore

**Keywords:** endoscopic gastrostomy

## Abstract

**Introduction.** Tumour cell implantation is a rare complication in patients with head and neck cancers, who have undergone percutaneous endoscopic gastrostomy (PEG) tube placement. It has not been reported in patients who underwent a PEG insertion via the radiological or introducer technique. We describe a novel case presentation of metastatic disease in a patient who underwent PEG placement via the introducer (Russell) technique which, to the best of our knowledge, has not not previously been described.

**Case presentation.** The patient was a 37-year-old Malay woman who developed metastatic squamous cell carcinoma deposits in her stomach and liver one month after a gastrostomy tube was removed following the completion of treatment for oropharyngeal carcinoma.

**Conclusion.** Previous authors have advocated the use of alternative PEG insertion technique apart from the ‘pull’ technique to minimise the risk of tumour implantation from head and neck cancers. Our case report suggests that this risk is not totally eliminated when the PEG tube is inserted via the introducer technique.

## BACKGROUND

Percutaneous endoscopic gastrostomy (PEG) is a commonly used method of enteral nutrition in patients with head and neck cancers. It has been shown to be more efficacious, compared with enteral nutrition via a naso-gastric tube [1]. The ‘Ponsky Pull’ technique was first introduced by Gauderer *et al.* in 1980 and is today the most commonly used method of gastrostomy tube placement [2].

A unique complication of PEG insertion in patients with head and neck cancers is that of tumour seeding and stromal metastases to the abdominal wall and viscera, especially the stomach. It was first described by Preyer and Thul in 1989 [3]. In a review of all 44 known reported cases, Cappell reported that strong risk factors for stomal metastases included primary pharynx-oesophageal cancer, squamous cell histology, less differentiated tumour, large size and advanced cancer stage, PEG placement by the Pull technique, untreated primary cancer with local recurrence and time >3 months after PEG insertion [4].

The possible pathogeneses of stomal metastases were postulated to be direct seeding, haematogeneous spread and shedding of tumour cells into the gastrointestinal tract [5–7]. However, it should be noted that, without PEG insertion, gastric metastasis from head and neck cancers is exceedingly rare. As both the ‘pull’ as well as the ‘push’ technique potentially expose the tumour to endoscope and guide wire during their trans-oral passage, it has been recommended by several authors that the use of direct endoscopic introducer technique (Russell’s technique) or insertion under radiological guidance may help to avoid this dreaded complication [4, 7, 8].

We believe that our case report is the first report of a head and neck cancer patient who suffered intra-abdominal metastases following PEG insertion using the Russell Introducer technique.

## CASE PRESENTATION

A 37-year-old woman with a history of systemic lupus erythematosus (SLE) and temporal lobe epilepsy was referred from a dentist to our hospital for right mandibular swelling. Further biopsy and radiological work-up showed a stage T2N0M0 squamous cell carcinoma (SCC) in the oropharynx. A PEG tube was inserted via the Russell ‘introducer’ technique prior to surgical and radiotherapeutic intervention, in anticipation of feeding difficulties during her treatment.

In the Russell ‘introducer’ technique, a diagnostic gastroscopy was first performed to ensure that the stomach was free of lesions. A suitable site was then identified for insertion of the PEG in the gastric wall. The insertion site of the PEG on the anterior abdominal wall was identified by transillumination of the gastric wall using the endoscope. Gastropexy T-fasteners were then secured to the wall under direct endoscopic guidance. An 18G large bore needle was inserted through the anterior abdominal wall and a guide wire passed through this needle into the stomach. The large-bore needle was then removed and the puncture site was serially dilated to allow the subsequent insertion of the gastrostomy tube. The final position of the tube was then checked and the tube flushed smoothly before the conclusion of the procedure.

**Figure 1.**
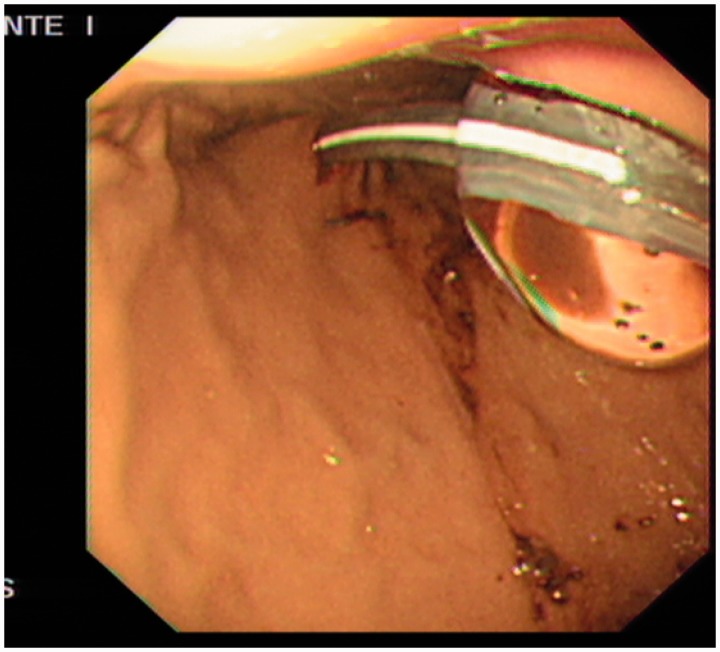
Stomach at time of PEG insertion.

Endoscopy showed a healthy stomach with no active lesions (Figure 1) and a PEG tube was inserted using the Russell Introducer technique, using the Kimberly-Clark MIC-G20 set. The patient underwent a curative surgical resection of the oropharyngeal tumour with selective neck lymph node dissection, followed by adjuvant radiotherapy. Histological examination of the resected specimen showed well to moderately differentiated SCC with clear resection margins. All lymph nodes harvested during surgery were found to be free from SCC. Upon completion of treatment, the PEG was removed 3 months later.

**Figure 2.**
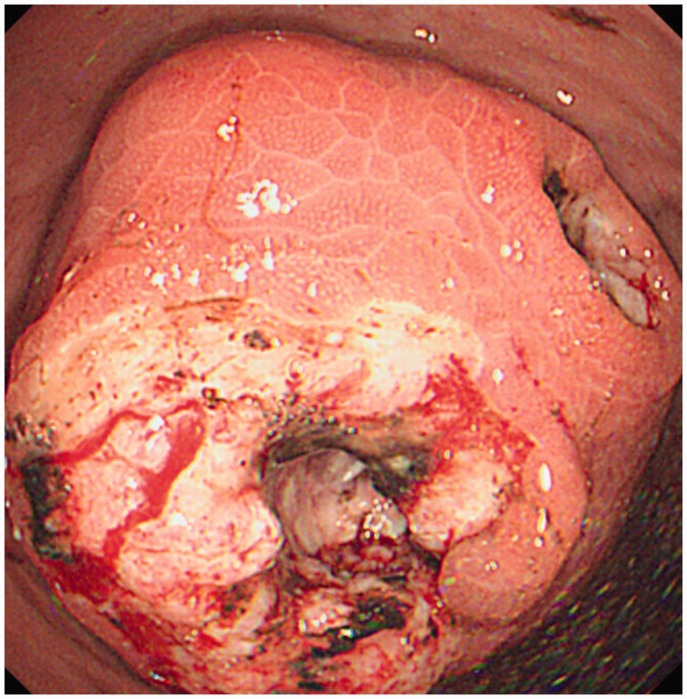
Tumour mass at PEG insertion site.

The patient subsequently presented a month later to the Emergency department with weight loss, abdominal pain and melaena. Upon further questioning, she had been having left upper quadrant abdominal pain since the PEG was inserted but it had progressively worsened since the tube was removed. Externally, the orifice at the site of insertion had healed well. Endoscopy revealed a friable 5–6 cm bulky tumour (Figure 2) along the greater curve of the stomach over the PEG insertion site with two large necrotic ulcers lying over the mass. This was confirmed histologically to be a moderately differentiated SCC. A computed tomography (CT) scan revealed an exophytic mass involving the greater curvature of the stomach with metastases to the liver.

The patient subsequently underwent palliative radiotherapy for treatment of her metastatic squamous cell carcinoma.

## DISCUSSION

Apart from the ‘Ponsky Pull’ method, where the PEG tube is pulled in through the oropharynx and oesophagus [2], the ‘push-over-wire’ and ‘introducer’ endoscopic methods have been described by Sacks and Russell, respectively [9, 10]. To date, less than 50 cases of stomal site metastases have been reported. All had PEG placement with the pull technique, except for two who underwent a ‘push-over-wire’ and operative gastrostomy, respectively [4].

In our patient, we had deliberately used the introducer technique to obviate contact between the tumour and inserted tube or wire. Also, the primary lesion was resected at an early stage. Our patient did not seem to be at particularly high risk of suffering stomal metastases, given that her tumour was at a fairly early stage and was moderately well-differentiated with no evidence of lymphatic or haematogenous spread at the time of resection.

We postulate that it is possible that long-term suppression of the gastric acid by proton pump inhibitors, which are frequently prescribed post-PEG, results in a hypo-acidic environment that allowed tumour growth. Furthermore, it is known that injured tissue (in this case, the PEG insertion site) is more susceptible to metastatic tumour growth and embolization [11]. The common practice of trans-illumination with the gastroscope tip pressed against the mucosa theoretically might seed cells onto the mucosa, which would later be incised, and this practice should be discouraged. Furthermore, the patient’s SLE could also have rendered her immune-compromised, making her more susceptible to tumour growth following implantation: further studies are needed to clarify whether this is a risk factor.

Previous authors have advocated the introducer technique to prevent implantation metastases, but we believe our case illustrates the fact that PEG insertion via the Russell technique does not eliminate the risk of direct tumour seeding. Possible explanations for the above case include occurrence of tumour seeding due to the gastroscope tip implanting cells directly into the gastric wall during transillumination or a result of the settling of swallowed tumour cells into the fresh gastric wound, which would have caused the metastases to occur regardless of the method of PEG insertion. Patients with head and neck cancers undergoing PEG insertion, regardless of the stage of neoplastic disease, will need to be counselled about—and accept the possible risk of—visceral and abdominal wall metastases. We would also encourage operators to avoid passage of the gastroscope deep into the stomach and omit the transillumination step to avoid inadvertent seeding of the primary tumour to the PEG wound site.

**Conflict of interest:** none declared.

## References

[1] Sadasivan A, Faizal B, Kumar M (2012). Nasogastric and percutaneous endoscopic gastrostomy tube use in advanced head and neck cancer patients: a comparative study. J Pain Palliat Care Pharmacother.

[2] Gauderer MW, Ponsky JL, Izant RJ (1980). Gastrostomy without laparotomy: a percutaneous endoscopic technique. J Pediatr Surg.

[3] Preyer S, Thul P (1989). Gastric metastasis of squamous cell carcinoma of the head and neck after percutaneous endoscopic gastrostomy: report of a case. Endoscopy.

[4] Cappell MS (2007). Risk factors and risk reduction of malignant seeding of the percutaneous endoscopic gastrostomy track from pharyngoesophageal malignancy: a review of all 44 known reported cases. Am J Gastroenterol.

[5] Coletti D, Genuit T, Ord R, Engroff S (2006). Metastasis to the percutaneous endoscopic gastrostomy site in the patient with head and neck cancer: a case report and review of the literature. J Oral Maxillofac Surg.

[6] Oakley RJ, Donnelly R, Freeman L (2009). An audit of percutaneous endoscopic gastrostomy insertion in patients undergoing treatment for head and neck cancer: reducing the incidence of peri-operative airway events by the introduction of a tumour assessment protocol. Ann R Coll Surg Engl.

[7] Ananth S, Amin M (2002). Implantation of oral squamous cell carcinoma at the site of a percutaneous endoscopic gastrostomy: a case report. Br J Oral Maxillofac Surg.

[8] Thakore JN, Mustafa M, Suryaprasad S, Agrawal S (2003). Percutaneous endoscopic gastrostomy associated gastric metastasis. J Clin Gastroenterol.

[9] Sacks BA, Vine HS, Palestrant AM (1983). A nonoperative technique for establishment of a gastrostomy in the dog. Invest Radiol.

[10] Russell TR, Brotman M, Norris F (1984). Percutaneous gastrostomy. A new simplified and cost-effective technique. Am J Surg.

[11] Murthy SM, Goldschmidt RA, Rao LN (1989). The influence of surgical trauma on experimental metastasis. Cancer.

